# Tranexamic acid can reduce blood loss and improve visibility in otological surgeries: a systematic review and meta-analysis of randomised controlled trials

**DOI:** 10.1017/S0022215125103599

**Published:** 2025-12

**Authors:** Piotr Domaszewski, Ayman Khatib, Brandon Goodwin, Sami Dakhel, Gabrianna Andrews, Adrianna Hekiert, Julia Rangel

**Affiliations:** 1 Department of Medicine, Rowan University School of Osteopathic Medicine, Stratford, NJ, USA; 2 Labyrinth - Future Osteopathic Scholars in Otolaryngology, Manasquan, NJ, USA; 3 Futures Forward Research Institute, Toms River, NJ, USA; 4 ENT and Allergy Associates LLP, Bridgewater, NJ, USA

**Keywords:** mastoiditis, neuro-otology, otitis media, otitis media with effusion

## Abstract

**Objectives:**

The objective of the study was to determine the effect of tranexamic acid in ear surgeries on duration of surgery, intra-operative blood loss, visibility and mean arterial pressure (MAP).

**Methods:**

A systematic review and meta-analysis were conducted following the 2020 PRISMA guidelines. Five databases were used (PubMed, Cochrane, Scopus, Web of Science and Embase). A search yielded 73 articles: 31 were duplicates and 42 were screened for by two authors. A standardised mean difference (SMD) was calculated to measure the effect size across studies.

**Results:**

The search yielded five final studies with ear procedures including tympanoplasty, atticotomy, mastoidectomy, ossiculoplasty, stapedotomy, tympanotomy and microscopic modified radical mastoidectomy. Tranexamic acid reduced duration of surgery (standardised mean difference = -3.82; *p* = 0.38), intra-operative blood loss (standardised mean difference = -19.64; *p* < 0.05) and mean arterial pressure (standardised mean difference = -2.88; *p* < 0.05).

**Conclusion:**

This meta-analysis demonstrated that tranexamic acid reduced bleeding and mean arterial pressure that were both statistically significant, while the reduction in duration of surgery was statistically insignificant. All studies reported better visibility.

## Introduction

Otologic surgical procedures vary widely and address various pathologies such as chronic ear infections, hearing improvement, structural repair and alleviating otorrhea.^1^ Procedures such as tympanoplasties and mastoidectomies are often essential for restoring ear function but can present significant challenges. Endoscopic surgery, for instance, has gained popularity in recent years due to its minimally invasive nature and enhanced magnification and imaging capabilities that provide superior visualisation of the surgical field.^2^ However, otologic surgeries present unique difficulties even for the most experienced surgeons due to the ear’s complex anatomy which can be easily obscured by minimal bleeding.^3^

The risk factors involved with otologic surgery include but are not limited to: pain, bleeding, infection, graft failure, recurrence of treated pathology and worsening of symptoms such as hearing, tinnitus, dizziness and facial nerve palsy.^4^^–^^6^ As the success of ear surgeries often relies on the ability to maintain clear visibility throughout the duration of the procedure, complications such as excessive bleeding can compromise surgical outcomes, especially for more novice surgeons.^3^^,^^7^ Several haemostatic agents such as adrenaline, adrenaline combined with lidocaine and absorbable gelatine sponges have been used to control intra-operative bleeding and improve visibility in otologic surgery.^8^^–^^10^ Lidocaine and adrenaline are frequently jointly administered, offering notable benefits, but also carry minor associated risks. While generally benign, both can potentially cause cardiovascular complications such as arrhythmia and tachycardia.^11^ Controlled hypotension is an additional tool used to reduce intra-operative bleeding; however, it carries a risk especially in patients with underlying cardiovascular disease. It is imperative to achieve a careful balance between optimal haemostasis with patient safety.^12^^–^^15^

Another proposed mechanism to reduce peri-operative bleeding is the use of intravenous tranexamic acid (TXA), a synthetic antifibrinolytic agent, which has shown success to date in various surgical subspecialties, such as cosmetic, orthopaedic and trauma surgery as well as obstetrics.^16^^–^^19^ TXA is a synthetic derivative of lysine that works by inhibiting the breakdown of fibrin clots by irreversibly blocking plasminogen’s binding sites, hence preventing plasmin activation and ultimately reducing bleeding.^20^

Despite its widespread use, limited research specifically examines the effects of TXA in otologic surgeries. Preliminary studies suggest that TXA may help reduce operative time and improve haemostasis, though its effects on mean arterial pressure (MAP) and overall surgical outcomes remain unclear, warranting further research.^21^^,^^22^

Given the lack of comprehensive reviews on the subject, this systematic review and meta-analysis aims to determine the efficacy of TXA in ear surgeries, specifically its impact on operative time, bleeding control and MAP. This study builds upon previous narrative reviews, providing a more detailed analysis of the use of TXA that could help improve surgical practices and outcomes in the field of otology.

## Methods

### Systematic review and meta-analysis

We performed a systematic review and meta-analysis of the literature to identify studies describing outcomes in patients, who received TXA pre-operatively during ear surgeries. Our primary outcomes included duration of surgery (DOS), MAP at 30 minutes, intra-operative blood loss (IOBL) and visibility scores. Secondary outcomes included complications, heart rate (HR) and recovery time. We prepared a detailed protocol about the inclusion and exclusion criteria, search strategy and statistical methods. The PRISMA (Preferred Reporting Items for Systematic Reviews and Meta-Analysis) guidelines were adhered to.^23^ Institutional Review Board (IRB) approval was not required since our review only included randomised controlled trials.

### Literature search strategy

**Figure 1.**
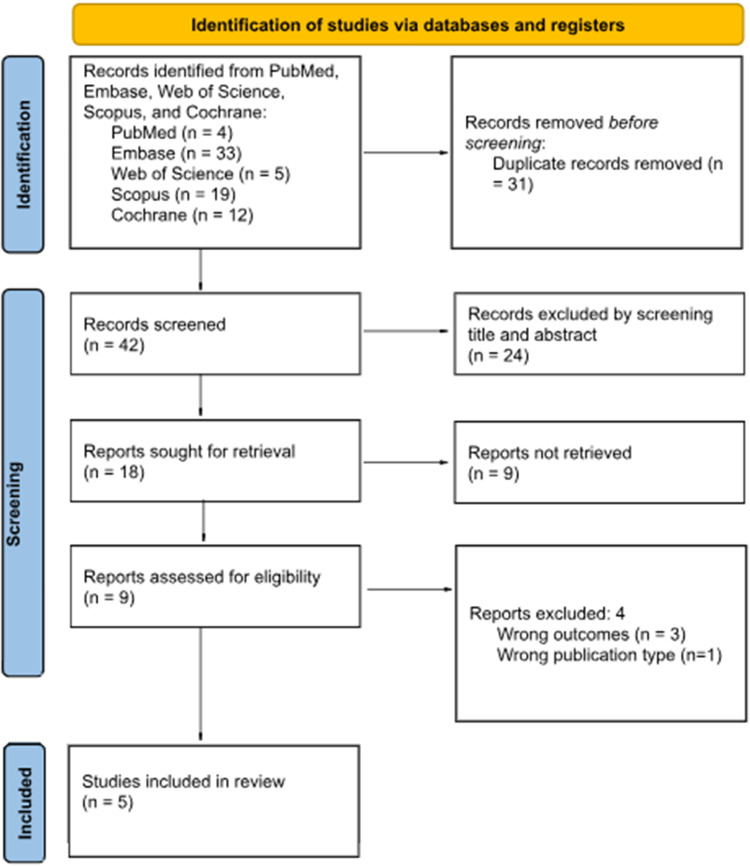
PRISMA flow diagram of how studies were selected.^23^ PRISMA = Preferred Reporting Items for Systematic Reviews and Meta-Analysis.

Electronic searches were performed using PubMed, Cochrane Library, Embase, Scopus and Web of Science databases. All published articles and abstracts were searched from their inception to 10 August 2024 and narrowed for English language literature. A detailed search strategy was developed using a combination of the following official MeSH terms, including tranexamic acid, tympanoplasty, myringotomy and stapedectomy, along with non-official MeSH terms ear surgery and ossiculoplasty. Two investigators (P.D., B.G.) performed the literature search. After duplicate articles were removed, all titles and abstracts were screened initially for inclusion and exclusion criteria. Relevant articles were identified, and two reviewers independently examined full texts (P.D., B.G.) to eliminate selection bias. A third reviewer (A.K.) resolved any conflicts regarding article inclusion. Full texts were then screened for and included based on the inclusion criteria. Search results are displayed in Figure 1.

### Inclusion and exclusion criteria

Our systematic review included studies that met our inclusion criteria: patients aged 18 or older who underwent any ear procedure including myringotomy, tympanoplasty, stapedectomy, ossiculoplasty, mastoidectomy or endoscopic ear surgery; with the intervention of TXA through any route of administration; studies with our primary outcomes of DOS, IOBL, visibility scores and MAP; finally, only randomised controlled trials (RCTs) were included.

Our study excluded studies based on the following criteria: non-RCT studies including case reports, non-randomised clinical trials, letters, observational studies, international surveys and retrospective reviews; studies that did not use TXA for haemostasis; patients who were under the age of 18; patients who did not undergo an otologic surgery/procedure; animal studies; and patients with underlying blood- and clotting-related disorders including haemophilia, congenital factor V and VIII deficiencies and hereditary haemorrhagic telangiectasia.

### Data extraction and statistical analysis

Two independent reviewers extracted data from the five included studies. The following data were extracted: title, author, year of publication, sample size, DOS, MAP at 30 minutes, IOBL in mean ± SD and visibility scores. IOBL was measured in millilitres (ml) by a suction chamber, by weighing used sponges and via specific scoring systems. Quantitative meta-analysis was performed for all outcomes and reported as standardised mean differences (SMDs) with the random effects model to account for heterogeneity. R-Studio was used for data analysis. Other information regarding mean patient age and mean dose of TXA given pre-operatively was collected in Tables 1 and 2. Visibility scores, HR at 30 minutes, surgeon satisfaction and recovery time were assessed qualitatively.

**Table 1.** Overview of TXA and control group for each study

Author	TXA	Control	Sample size	Procedure	Study type
Das *et al*.^24^	IV TXA 10 mg/kg 30 minutes before surgery and IV TXA 5 mg/kg every hour	Normal saline	50	Tympanoplasty, atticotomy, mastoidectomy, ossiculoplasty, stapedotomy	Double-blind, randomised, placebo controlled trial
Hamed and Hamed^26^	Topical 1000 mg of TXA diluted in 200 ml of normal saline (0.9%)	Topical adrenaline 1 mg diluted in 200 ml normal saline (0.9%)	60	Tympanotomy	Randomised double-blind trial
Zhang *et al*.^21^	IV TXA (1 g in 20 ml of normal saline)	20 ml of normal saline administered for 10 min	56	microscopic modified radical mastoidectomy	Randomised, double-blind, controlled trial
Ziaei *et al*.^25^	IV TXA 10 mg/ kg before procedure	Normal saline	70	Mastoidectomy	Randomised double-blind clinical
Aghadavoudi *et al*.^27^	IV TXA 10 mg/kg before surgery and remifentanil (0.1 μg/kg/min) during surgery	Remifentanil (0.2 μg/kg/min) during the surgery	69	Mastoidectomy	randomised control trial

Abbreviations: IV = intravenous; TXA = tranexamic acid.

**Table 2.** Demographics of each study

Author	Age (mean, standard deviation)		Sex (male, female)	
	TXA	Control	TXA	Control
Das *et al*.^24^	28.96 ± 16.9	28.06 ± 6.8	15, 10	14, 11
Hamed and Hamed^26^	32 ± 9.7	35 ± 10.9	14, 16	16, 14
Zhang *et al*.^21^	44.96 ± 12.62	46.21 ± 11.52	14, 14	10, 18
Ziaei *et al*.^25^	32.87 ± 7.9	33.20 ± 6.25	20, 14	18, 17
Aghadavoudi *et al*.^27^	40.8 ± 7.79	33.4 ± 6.23	21, 13	19, 16

Abbreviation: TXA = tranexamic acid.

### Assessment of bias and study heterogeneity

Since only RCTs were included in the present study, two authors performed risk of bias assessment (P.D., B.G.); A.K. was the third reviewer, if needed. The risk of bias was appraised using version 2 of the Cochrane risk-of-bias tool for randomised trials (RoB 2). Each study was weighted at an equal level in the assessment. The data are presented in a traffic-light plot in Figure 4 and a summary chart in Figure 5. Inclusion criteria in all studies were carefully reviewed to identify any treatment bias. The results were reviewed by the senior reviewer (P.D.). Funnel plots for primary outcomes, that is, DOS, MAP and IOBL were created. Visibility scores were reviewed qualitatively. The significance of plot symmetry was calculated with the Egger’s test using a mixed-effects meta-regression model. Study heterogeneity was assessed using the Cochran Q and I^2^ test. Sensitivity analysis was performed for outcomes with significant outliers, by removing the outlier.

## Results

After the primary screening of the title and abstract, 24 out of 42 records were excluded. The studies were excluded due to surgical techniques unrelated to the ear, wrong study design, such as retrospective and prospective cohort or reviews case reports, or lack of TXA application. Of the 18 remaining studies, 9 were abstracts only and, therefore, had to be excluded from the systematic review. After full-text review of the remaining nine studies, our systematic review and meta-analysis included five final studies. Four studies were removed from final inclusion as they lacked the outcomes of interest or involved patients having underlying blood related disorders. DOS analysis was extracted from four studies (236 patients),^21^^,^^24^^–^^26^ IOBL from three studies (199 patients)^25^^–^^27^ and MAP from three (189 patients).^24^^,^^25^^,^^27^

### Effects of intervention (meta-analysis)

**Figure 2.**
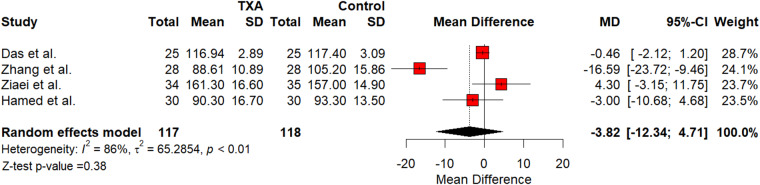
Forest plot of TXA’s impact on DOS. CI = confidence interval; DOS = duration of surgery; SD = standard deviation; TXA = tranexamic acid.

**Figure 3.**
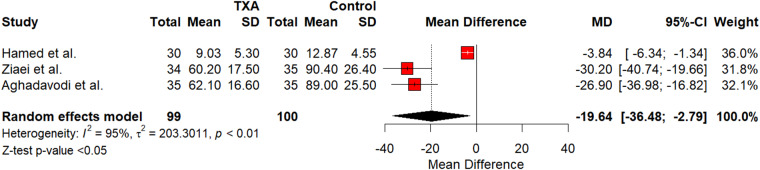
Forest plot of TXA’s impact on intra-operative bleeding volume. CI = confidence interval; SD = standard deviation; TXA = tranexamic acid.

**Figure 4.**
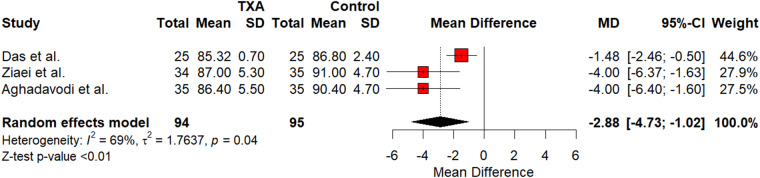
Forest plot of TXA’s impact on MAP. CI = confidence interval; MAP = mean arterial pressure; SD = standard deviation; TXA = tranexamic acid.

Meta-analysis of DOS showed a small reduction without statistical significance as shown in Figure 2 (SMD = -3.82; 95 per cent confidence interval [CI] [-12.3426; 4.7120]; *p* = 0.38). The meta-analysis showed heterogeneity for DOS (I^2^ = 86 per cent; *p* < 0.01). IOB was shown to decrease that was statistically significant, as shown in Figure 3 (SMD = -19.638; 95 per cent CI [-36.4827; -2.7933], I^2^ = 0.22; *p* < 0.01). Heterogeneity for IOBL was (I^2^ = 95 per cent; *p* < 0.01). TXA showed a statistically significant decrease in MAP as shown in Figure 4 (SMD = -2.88; 95 per cent CI [-4.7336; -1.0195]; *p* < 0.01). Heterogeneity for MAP was (I^2^ = 69 per cent; *p* < 0.05). Funnel plots were generated for all outcomes showing symmetry and lack of publication bias. Publication bias was further tested using Egger’s regression analysis, indicating lack of publication bias for DOS (*p* = 0.18), intra-operative bleeding (*p* = 0.67) and MAP (*p* = 0.83).

### Risk of bias assessment

**Figure 5.**
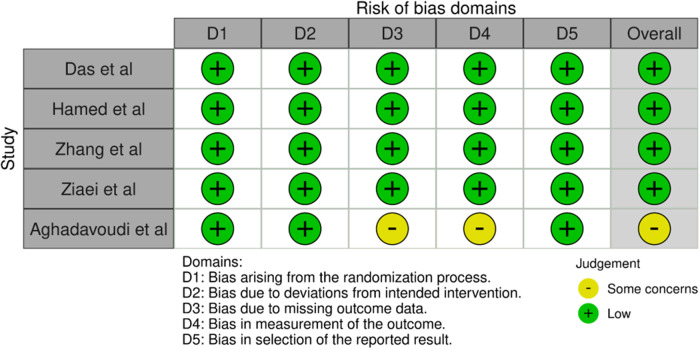
RoB-2 traffic light plot.^34^ RoB-2 = Cochrane risk-of-bias tool for randomised trials.

**Figure 6.**
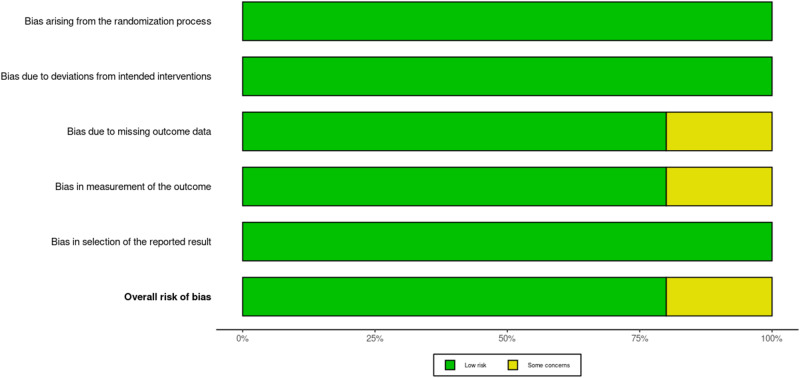
RoB-2 summary plot.^34^ RoB-2 = Cochrane risk-of-bias tool for randomised trials.

Risk of bias is displayed in a traffic-light plot in Figure 5 and a summary chart in Figure 6. Aghadavoudi *et al*. had some concerns for domains 3 and 4 of RoB-2. There was some concern for domain 3, or bias due to missing outcomes, due to the loss to follow-up of one patient in their TXA group. Domain 4 or bias in the measurement of outcomes also held concern since surgeon satisfaction was not specified clearly. The remaining studies had low bias.

### Qualitative analysis

Surgeon satisfaction, HR and recovery time were outcomes that were part of our systematic review; however, they could not be analysed quantitatively due to only two studies reporting each of these outcomes. Surgeon satisfaction was included in Zhang *et al*. and Aghadavodi *et al*. both using different metrics for satisfaction. Zhang *et al*. used 0 to 10 Numeric Rating Scale, 0 being worst and 10 being best.^21^ For their TXA group, satisfaction was rated with a mean and standard deviation of 7.82 ± 0.55 and 6.50 ± 0.64 for their control group (*p* < 0.001). Aghadavodi *et al*. measured surgeon satisfaction by weak, moderate, good or excellent. A total of 40 per cent of the surgeons in the control group reported excellent satisfaction, while 82.4 per cent reported excellent satisfaction in the TXA group.^27^ HR at 30 minutes into the procedures was recorded in Das *et al*. and Ziaei *et al*. HR in beats per minute was 89.36 ± 0.707 and 89.56 ± 2.64 in Das *et al*. for TXA and control groups respectively (*p* = 0.776).^24^ In Ziaei *et al*. HR was 74.3 ± 6.9 for the TXA group and 77.8 ± 4.0 for the control group (*p* = 0.712).^25^ Recovery time in minutes was recorded in Ziaei *et al*. and Hamed and Hamed.^26^ Recovery time in the TXA group of Ziaei *et al*. was 47.8 ± 6.6 and 44.8 ± 8.1 for their control group (*p* = 0.227). In Hamed and Hamed, recovery time was 9.9 ± 3.2 and 10 ± 2.0 for TXA and control groups, respectively (*p* = 0.583).

## Discussion

Excessive bleeding is a challenging issue for otologic surgeons as it obscures visibility in the operative field. TXA is a haemostatic agent that has been successfully used by various surgical specialties to improve intra-operative haemostasis.

TXA use in otologic surgery remains relatively unexplored with merely a few randomised controlled trials on the subject. Our systematic review and meta-analysis analysed and quantified all the available trials for IOBL, DOS and MAP to evaluate its effectiveness in improving visibility in ear surgeries. Patients were excluded from each of the individual studies if they had the following contraindications for TXA: active middle-ear infections, signs of middle ear inflammation, patients on anticoagulation therapy, bleeding or coagulation disorders, heparin use within two days of surgery or aspirin use within seven, pregnancy or lactation, colour blindness, thromboembolic disorders, hypertension, seizures or hepatic, renal or cardiac failure.^21^^,^^24^^–^^27^

### IOBL

The main outcome of our meta-analysis was blood volume, or IOBL. Out of the five studies used in our analysis, only three recorded bleeding volume or IOBL in ml.^25^^–^^27^ Analysis of these three showed that TXA greatly reduced bleeding that was statistically significant (SMD = -19.638; *p* < 0.01). This suggests that TXA effectively reduces bleeding during otologic surgeries. These results are consistent with TXA’s mechanism of action. In their meta-analysis on TXA use in nasal surgery, Ping *et al*. used a different method of analysis but also indicated significant reductions in blood loss.^28^ One of the RCTs used in our analysis was the only trial that compared TXA to adrenaline. Hamed and Hamed evaluated IOBL every 15 minutes during tympanotomy and found that, at each time, TXA resulted in statistically significant decrease in blood loss than adrenaline.^26^ Das *et al*. was one of the studies that did not report values for IOBL due to their primary outcome being visibility during surgery; however, they found that TXA did significantly improve visibility due to controlling blood loss.^24^ Similarly, the primary outcome for Zhang *et al*. was visibility, and they did not record values on blood loss since modified radical mastoidectomy procedures result in less blood loss.^21^ They reported significantly improved visual fields in their TXA group compared with their control. With effective haemostasis in otologic procedures, surgeons gain improved visibility of the operative field, which could minimise the risk of iatrogenic complications.

### DOS

Operative time or DOS was another main outcome of our meta-analysis. Four of our studies reported DOS in minutes. The analysis for DOS showed a small reduction that was statistically insignificant (SMD = -3.82; *p* = 0.38). Although it does not suggest a conclusive clinical effect, it may warrant further investigation in future studies. Ping *et al*. also showed similar insignificant results for DOS.^28^ This could be due to each randomised controlled trial’s different procedures and surgeries. The results for DOS for Hamed and Hamed were also insignificant but showed that TXA had shorter DOS compared with their adrenaline group.^26^ Of the four trials used in the analysis for DOS, only Ziaei *et al*. had an increase in DOS.^25^ In Ziaei *et al*. all patients in their study underwent mastoidectomies. In Das *et al*. three patients underwent mastoidectomies, and their pooled data for DOS showed a statistically insignificant reduction.^24^ In Zhang *et al*., all patients underwent microscopic modified radical mastoidectomy, and they showed a statistically significant reduction in DOS.^21^ Aghadavodi *et al*. was one of the studies used in our systematic review, however, it was not used in the analysis for DOS since they did not report it. Although not statistically significant, TXA is at least as good as previous methods since it does not result in an increase in DOS. Heterogeneity for DOS was high (I^2^ = 0.86), most likely due to Zhang *et al*. In their study, Zhang *et al*. used a different dose of TXA compared to the other studies.^21^

### MAP

MAP was the final comparable outcome for the study. Of the five studies used, only three reported MAP at 30 minutes into the procedures.^24^^,^^25^^,^^27^ Analysis showed that the reduction that TXA had on MAP was statistically significant (SMD = -2.88; *p* < 0.01). This small reduction in MAP may or may not be desirable, however it should not raise the concern for the haemodynamic stability of patients undergoing ear surgeries, as none of the patients in the studies experienced instability with TXA use. Hamed and Hamed was not used in the analysis of MAP; however, they did display a chart comparing mean blood pressure for their TXA and adrenaline groups.^26^ The adrenaline group sustained higher mean blood pressures at 30 minutes than TXA did. A recent systematic review suggested similar findings. They hypothesised that the potential benefit of a low MAP during such procedures may be due to reduced sympathetic activity contributing to haemostasis, or to TXA itself having a direct effect on vascular tone.^29^

### Visibility

Three studies included visibility scoring systems; however, they all used different systems. Despite the different scoring systems used, they all have the same general elements. Das *et al*. used the Das and Mitra scoring system on a 0–5 scale, where grade 0 was given if there was no bleeding and grade 5 if there was severe bleeding. Hamed and Hamed used the Boezaart scoring system also on a 0–5 scale, where grade 0 was given if there was no bleeding and grade 5 for severe bleeding. Lastly, Zhang *et al*. used the Modena scoring system that gives a score from 1to 5, where 1 is no bleeding and 5 is bleeding that prevents procedure. For Das *et al*., the Das and Mitra median scores were the same between the TXA and control groups.^24^ However, the TXA included scores that ranged lower than the control group. This was found to be statistically significant. For Hamed and Hamed, TXA had lower and better Boezaart scores at all time points, which were all statistically significant.^26^ In Zhang *et al*. TXA had significantly lower Modena scores than their control.^21^ Although no statistical analysis could be done on visibility, all randomised controlled trials reported better visibility during surgery due to haemostasis from TXA. A narrative review on how TXA improved visibility during such procedures also had similar findings, concluding that the reduction in intra-operative bleeding allowed for improved visibility.^30^

### Surgeon satisfaction, HR and recovery time

Surgeon satisfaction, HR and recovery time were only reported by two studies, and therefore not included in the meta-analysis. Surgeon satisfaction for both Zhang *et al*. and Aghadavodi *et al*. was higher in TXA groups.^21^^,^^27^ This is most likely due to reduced bleeding, improved visibility and shorter DOS. Das *et al*. and Ziaei *et al*. both found insignificant results for HR at 30 minutes, as both were in normal physiological ranges.^24^^,^^25^ Lastly, recovery time in Ziaei *et al*. was longer in the TXA group compared to the control, but this was not statistically significant. The TXA group in Hamed and Hamed reported a reduction in recovery time compared to control, but this was not statistically significant.^26^

### Adverse effects

Some of the adverse effects of TXA include headache, seizures, abdominal pain, nausea, vomiting, diarrhoea, pulmonary embolism, altered colour vision, deep vein thrombosis and anaphylactic shock.^31^ Our systematic review showed that Aghadavoudi, Das and Ziaei did not report any outcomes on adverse events or complications.^24^^,^^25^^,^^27^ Hamed and Hamed found no complications in their TXA group.^26^ Zhang *et al*. also had no adverse events in 12 months of follow-up in their TXA group.^21^ A systematic review concluded that TXA was safe and resulted in no adverse events in tonsillectomies, but the effect of TXA was unclear in major head and neck surgeries.^32^ Taeuber *et al*. determined that TXA administered intravenously did not increase the risk for thromboembolic events in neurological procedures.^33^ While TXA can result in several adverse effects, they typically occur in patients with comorbidities and especially cardiovascular diseases. None of the studies reported facial nerve injuries.

### Limitations

One limitation of our meta-analysis is that it only included five studies, and not all five studies reported the same outcomes, adding to heterogeneity. The lack of identical otologic procedures amongst the studies also constitutes part of the heterogeneity. Das *et al*. included patients undergoing tympanoplasty, atticotomy, mastoidectomy, ossiculoplasty and stapedotomy, while Hamed and Hamed only included patients undergoing tympanotomy.^24^^,^^26^ All studies had highly homogenous demographics between their TXA and control groups, except for Aghadavoudi *et al*. TXA dosage was relatively consistent amongst studies, except Hamed and Hamed used topical TXA while the remainder used TXA intravenously.^26^ Of the studies that used TXA intravenously, three of them used the same dose while Zhang *et al*. used 1 g of TXA in 20 ml of normal saline.^21^ Finally, the lack of post-operative audiometry, facial nerve outcomes and other intra-operative complications brings to question whether TXA improves clinical outcomes or avoids certain complications intra-operatively. Studies should be standardised to include and investigate such findings.

- Tranexamic acid (TXA) has been studied in surgical fields and has been shown to reduce blood loss
- TXA can cause complications such as nausea, vomiting, blood clots, seizures, headaches, diarrhea and visual disturbances
- TXA can improve the clinical outcomes of patients undergoing various surgeries
- Clinical relevance: This study analyzed if TXA improves visibility for surgeons and reduces complications such as blood loss, nausea and vomiting and if TXA affects the hemodynamic stability of patients undergoing otologic surgeries, and by how much

## Conclusion

Despite the small study pool, our systematic review and meta-analysis indicate that the use of TXA in otologic surgeries demonstrates promising advantages such as reduced intra-operative bleeding, decreased MAP as well as improved surgeon satisfaction due to enhanced visibility. TXA may also reduce operative time. While these results are encouraging, future studies should further explore other factors in peri-operative TXA administration such as dosage, optimal administrative route and timing. Additionally, more studies are needed to focus on the impact of TXA on surgical outcomes, including audiometry and facial nerve complications. More randomised controlled trials with unified protocols are needed to validate the findings and reduce variability.

## Supporting information

Domaszewski et al. supplementary material 1 Domaszewski et al. supplementary material

Domaszewski et al. supplementary material 2 Domaszewski et al. supplementary material

Domaszewski et al. supplementary material 3 Domaszewski et al. supplementary material

## Supplementary material

The supplementary material for this article can be found at https://doi.org/10.1017/S0022215125103599.
