# Supplementary material for: Tranexamic acid can reduce blood loss and improve visibility in otological surgeries: a systematic review and meta-analysis of randomised controlled trials
Source: J Laryngol Otol. 2025 Dec;139(12):1151–8. doi: 10.1017/S0022215125103599 (PMC12674988; doi:10.1017/S0022215125103599)
Supplement: Domaszewski et al. supplementary material 1 — Domaszewski et al. supplementary material [file S0022215125103599sup001.docx]

| **Score** | **Bleeding Description** |
| --- | --- |
| 1 | No bleeding |
| 2 | Bleeding easily controlled by suctioning, washing, or packing without any  significant modification or slowing of surgical procedure |
| 3 | Bleeding slowing surgical procedure |
| 4 | Most of the maneuvers dedicated to bleeding control |
| 5 | Bleeding that prevents every surgical procedure except those dedicated to  bleeding control |

**Table 6.** Modena bleeding scoring system used in Zhang et al. [21]
