# Supplementary material for: Tranexamic acid can reduce blood loss and improve visibility in otological surgeries: a systematic review and meta-analysis of randomised controlled trials
Source: J Laryngol Otol. 2025 Dec;139(12):1151–8. doi: 10.1017/S0022215125103599 (PMC12674988; doi:10.1017/S0022215125103599)
Supplement: Domaszewski et al. supplementary material 2 — Domaszewski et al. supplementary material [file S0022215125103599sup002.docx]

|  | **Hamed et al.** | | **Zhang et al.** | |
| --- | --- | --- | --- | --- |
|  | Control | TXA | Control | TXA |
| Nausea and Vomiting | 0 | 0 | 2 | 3 |
| Thromboembolic events | 0 | 0 | 0 | 0 |

**Table 1.** Reported postoperative complications
