# Supplementary material for: Tranexamic acid can reduce blood loss and improve visibility in otological surgeries: a systematic review and meta-analysis of randomised controlled trials
Source: J Laryngol Otol. 2025 Dec;139(12):1151–8. doi: 10.1017/S0022215125103599 (PMC12674988; doi:10.1017/S0022215125103599)
Supplement: Domaszewski et al. supplementary material 3 — Domaszewski et al. supplementary material [file S0022215125103599sup003.docx]

| **Author** | **Title** | **Reason for Exclusion** |
| --- | --- | --- |
| Al-Huniti 2022 | The Use of Liquid Tranexamic Acid for Pediatric Patients with Bleeding Disorders | Wrong population and patients had underlying blood disorders |
| Everberg 1977 | Plasma coagulation in myringoplasty and fascialis transplantation. | Primary outcomes of IOBL, DOS, visibility, and MAP are not included |
| Harris 1980 | Preoperative test of bleeding time in ear surgery | Primary outcomes of IOBL, DOS, visibility, and MAP are not included |
| Zhang 2024 | Efficacy and safety of intravenous tranexamic acid in microscopic modified radical mastoidectomy: A study protocol for a prospective, randomised, double-blind controlled trial | This was a trial registration for one of the studies used in our meta analysis, and did not provide the primary outcomes |
